# Impact on the *Leishmania mexicana* transcriptome due to knockout of genes encoding orthologs of methyltransferases involved in m1A and m5C mRNA modifications

**DOI:** 10.1186/s13071-025-06969-8

**Published:** 2025-07-31

**Authors:** Angela Moreira Bezerra, Ariely Barbosa Leite, Christian Robson de Souza Reis, João Luiz de Lemos Padilha Pitta, Suellen Rodrigues Maran, Nilmar Silvio Moretti, Danielle Maria Nascimento Moura, Antonio Mauro Rezende

**Affiliations:** 1 Department of Microbiology, Aggeu Magalhães Institute, Fiocruz Pernambuco, Recife, PE Brazil; 2 Department of Immunology, Aggeu Magalhães Institute, Fiocruz Pernambuco, Recife, PE Brazil; 3 Department of Microbiology, Immunology and Parasitology, UNIFESP, São Paulo, SP Brazil; 4 Department of Pathology and Microbiology, Faculty of Veterinary Medicine, Université de Montréal, Saint-Hyacinthe, Canada; 5 The Research Group on Infectious Diseases in Production Animals (GREMIP), Faculty of Veterinary Medicine, University of Montreal, Saint-Hyacinthe, Canada; 6 Institute of Biological Sciences, University of Pernambuco (ICB/UPE), Recife, PE Brazil; 7 Research Group of Biotechnology Applied to Pathogens, René Rachou Institute, Fiocruz Minas Gerais, Belo Horizonte, MG Brazil

**Keywords:** Chemical modification, m1A, m5C, Gene regulation, mRNA, *Leishmania mexicana*

## Abstract

**Background:**

Chemical modifications of mRNAs constitute an alternative mechanism for gene expression regulation, which involves proteins responsible for adding, recognizing and removing these modifications. While orthologs of enzymes involved in adding m1A (TRMT6/TRMT61A) and m5C (NSUN2) modifications are present in trypanosomatid species, a clear understanding of their biological role in these parasites is necessary.

**Methods:**

To shed light on this, we genetically manipulated the TRMT61A and NSUN2 protein-encoding genes in the *Leishmania mexicana* species using the CRISPR-Cas9 editing technique and analyzed the impact on cell growth and differentiation as well as the global gene expression profile.

**Results:**

Deletion of the genes investigated here caused changes in the normal pattern of *L. mexicana* differentiation, and functional analyses of differentially expressed genes in the mutants unveiled significant biological effects. For the TRMT61A gene, transcripts related to nucleotide metabolism, translation, protein folding and refolding were affected. For the NSUN2 genes, enrichment analysis indicated impacts on biological processes mostly related to nucleotide metabolism and DNA binding.

**Conclusions:**

Our findings provide insights into the role of these methyltransferases orthologs in the regulation of trypanosomatid transcriptome, contributing to our understanding of gene expression control in this parasite.

**Graphical Abstract:**

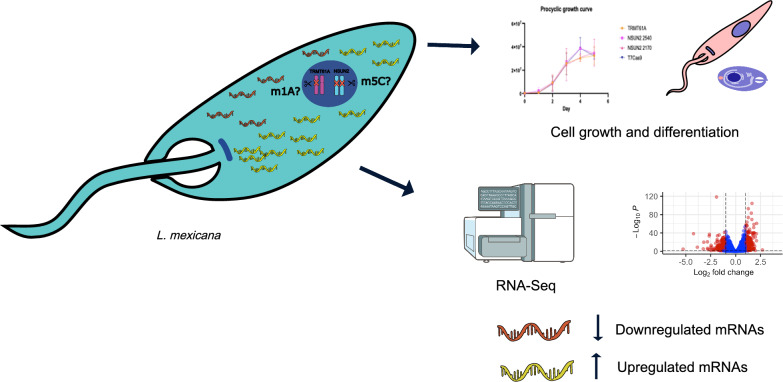

**Supplementary Information:**

The online version contains supplementary material available at 10.1186/s13071-025-06969-8.

## Background

In general, transcription is the main point of gene expression regulation in eukaryotes. However, despite recent evidence of identification of specific regulatory elements on coding regions of polycistronic mRNAs of trypanosomatids [1], for most of their genes, transcription initiation takes place in divergent strand switch regions (SSR), which have a shortage of promoters for RNA Pol II [2]. Thus, the gene expression regulation in these parasites mainly occurs at the post-transcriptional level by mRNA processing, degradation/stabilization and protein synthesis of specific mRNAs [3].

The regulatory mechanisms involved in the translation of mRNA molecules into proteins, specifically at the post-transcriptional level that controls RNA metabolism, e.g., expression patterns, subcellular localization, splicing and stability, are still not well understanding. Recent findings have shed light on the role of the dynamic chemical alterations of RNA nucleotide bases as crucial regulators of RNA metabolism, affecting all the steps of mRNA life [4–6]. More than a hundred of these modifications were identified in all types of RNAs (tRNA, rRNA, mRNA and small RNAs) in prokaryotes and eukaryotes, and all are known as epitranscriptomes [4, 7]. Therefore, we hypothesize that the epitranscriptome plays a pivotal role in gene expression regulation for the trypanosomatids as all their expression control lies in post-transcriptional biological events.

Among the RNA modifications that comprise the epitranscriptome of an organism, N1-methyladenosine (m1A) and 5-methylcytosine (m5C) can be highlighted by their abundance and number of studies [8, 9]. The effects of those modifications depend on the type and extent of transcripts modified [10]; their levels on the RNAs are regulated by a group of RNA-binding proteins, which can be broadly categorized into writers, the enzymes responsible for installing the modification; erasers, the enzymes responsible for removing the modification; and the readers, which recognize and bind to the modified RNA sequence leading to downstream effects [11].

The m1A was described for the first time in *Saccharomyces cerevisiae* tRNAmet predominantly at position 58, ensuring the proper folding of the tRNA molecule and preserving the stability of the tRNAmet [12]. This modification is also present in the structure of the mRNA, mainly in the 5’UTR, around the start codon [13]. The presence of this modification near the start codon suggests a possible relationship with translation initiation [14]. m1A is added to the RNA molecule by the heterodimer TRMT6/TRMT61A, where TRMT61A is a methyltransferase, while TRMT6 is the accessory subunit that binds to the mRNA, facilitating the insertion of the modification [15]. The removal of m1A is catalyzed by ALKBH1, ALKBH3, ALKBH7 and FTO, while four readers containing the YTH domain have been associated with the recognition of this modification (YTHDF1, YTHDF2, YTHDF3 and YTHDC1) [16, 17].

The m5C methylation is enriched in the 3' UTR region of mRNAs or near the translation initiation codon [18] in the human transcriptome. The presence of m5C impacts the stability and translation of the modified mRNA molecule [19]. The NSUN2 methyltransferase has been characterized as responsible for adding the m5C modification, while TET1 is implicated in its removal. ALYREF or and YBX1 have been described to be involved in the recognition of that modification [17, 20].

One of the first important pieces of evidence for the impact of mRNA modification on gene expression regulation in trypanosomatids came from the demonstration of the presence of m6A in the poly-A tails of variant surface glycoprotein (VSG) mRNAs in the bloodstream form of *Trypanosoma brucei* [21]. In addition, m6A is present in the different phases of the parasite’s life cycle, including the slender and stumpy forms. The distribution of m6A changes throughout these phases, and 47% of m6A methylated transcripts are unique to the stumpy form, suggesting that m6A is important in the regulation of these transcripts and the preparation for the parasite to exit the cell cycle [22]. Furthermore, previous work from our group, using bioinformatics tools, indicated the presence of orthologs for the writer's enzymes of m1A (TRMT6/TRMT61A), m5C (NSUN2) and ac4C (NAT10) [23]. Given the identification of potential homologous genes encoding proteins responsible for the addition of m1A and m5C modifications, we decided to assess the functional roles of those proteins that are candidates for influencing the epitranscriptome in trypanosomatids. Thus, the CRISPR-Cas9 genome editing system was applied to evaluate the impact of the deletion of the writer enzymes TRMT61A and NSUN2 on the global gene expression profile in *Leishmania mexicana*.

Our study demonstrates that TRMT61A or NSUN2 proteins are important for maintaining the regular patterns of cell differentiation during the parasite life cycle, and their deletions impact the global transcriptome. The deletion of the three genes investigated here decreased the rate of metacyclogenesis and altered the differentiation profile from promastigote to axenic amastigote. TRMT61A partial knockout caused changes in the expression levels of a larger set of genes, including some related to nucleotide metabolism, translation and protein folding and refolding. For NSUN2, the major changes observed were related to protein folding and refolding and metabolism and activity of nucleotides. Furthermore, the orthologs of those methyltransferases also seem to influence the levels of transcripts of multiple surface proteins of *L. mexicana*. Our findings reinforce that m1A and m5C modifications may play a role in the gene expression regulation process in *Leishmania*.

## Methods

### PCR amplification of DNA donors and sgRNA templates for CRISPR-Cas9

The DNA donors required for implementing the CRISPR-Cas9 technique were obtained through standard PCR reactions. The DNA donors were composed by sequences of the upstream and downstream intergenic regions (IR) of the target genes and the coding sequences of the resistance markers. The target genes were TRMT61A (ID: LmxM.28.2400) and two paralogs of NSUN2 (IDs: LmxM.36.2170 and LmxM.08.29.2540), which, for clarity, will be referred to here as NSUN2 2170 and NSUN2 2540, respectively. Therefore, the sequences of the primers (Additional file 1: Supplementary Table S1) were designed according to the knockout strategy available on the website www.leishgedit.net [24]. For the PCRs, 30 ng of the plasmids PGL2662, containing the resistance marker for blasticidin (*BSD*), and PGL2667, with a gene that confers resistance to puromycin (*PAC*), were used as DNA template. The reactions also included *Taq* DNA polymerase (5 U) (Invitrogen), dNTPs (125 μM), forward primer (2 μM), reverse primer (2 μM) and MgCl_2_ (25 mM) in a final volume of 40 µl. The PCR protocol consisted of an initial denaturation step at 94 °C for 3 min, followed by 35 cycles of denaturation at 95 °C for 30 s, annealing at 65 °C for 30 s, extension at 72 °C for 2 min and 15 s and a final elongation step at 72 °C for 7 min. Afterwards, the amplicons were confirmed by electrophoresis on 1% agarose gel.

To obtain the DNA fragments for sgRNA transcription, we also used the LeishGEdit tool (www.leishgedit.net). The primers generated (Supplementary Table S1) were used in PCR reactions with *Taq* DNA polymerase (5 U) (Invitrogen), dNTP (250 μM), forward primer (2 μM), reverse primer (2 μM), MgCl2 (25 mM) and 10× buffer. The PCR protocol included an initial denaturation at 95 °C for 30 s, followed by 35 cycles of denaturation at 95 °C for 10 s, annealing at 60 °C for 30 s and extension at 72 °C for 2 min and 15 s. The final extension step was performed at 72 °C for 10 min. Afterwards, the amplicons were checked using electrophoresis on 1% agarose gel.

### Cell culture and transfection

Promastigote cells of *L. mexicana* wild type (WT) (MHOM/GT/2001/U1103) and the transgenic cell line harboring the T7/Cas9 machinery [24] were cultivated at 28 °C in M199 medium pH 7.4, supplemented with 40 mM Hepes, 0.1 mM adenine, 0.0001% D-biotin and 4.62 mM NaHCO_3_, 10% fetal bovine serum (FBS), penicillin (50 U/ml) and streptomycin (50 µg/ml); 50 μg/ml of hygromycin was added to maintain the T7/Cas9 transgenes.

The transfections followed standard conditions as described elsewhere in [25]. Briefly, the two donor DNAs, each one with one resistance marker, were thermally sterilized at 94 °C for 5 min without any additional purification step. Afterwards, these PCRs products were mixed with a pellet of 1 × 10^7^ cells, 18 µl of sgRNA templates and Cytomix buffer in a final volume of 200 µl and then subjected to transfection with program X-001 of the Nucleofector Amaxa IIb (Lonza). The electroporated cells were transferred to a 25 cm^2^ bottle containing 10 ml M199 medium supplemented with 10% FBS and allowed to recover for 16 h. The following day, 20 µg/ml of blasticidin and 50 µg/ml of puromycin were added to the cultures for selection of the resistant parasites.

### Gene knockout confirmation

**Fig. 1:**
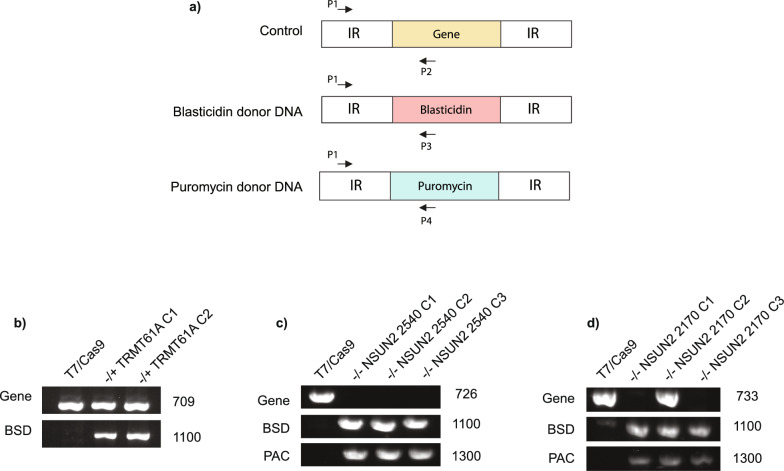
Confirmation of TRMT61A and NSUN2 knockout mutant cell lines. **a** Schematic representation of the PCR-based strategy used to confirm the substitution of endogenous target genes with the specific blasticidin (BSD) and/or puromycin (PAC) drug-selectable markers. The position of the primers used in the PCR reactions is indicated by arrows; IR = intergenic region. **b**–**d** Agarose gels of confirmatory PCR products of each cell line generated. **b** Fragments corresponding to the wild-type TRMT61A gene as well as the BSD cassette integration are shown. **c** and **d** Fragments corresponding to the wild type for NSUN2 coding genes (NSUN2 2540 and NSUN2 2170, respectively) as well as the BSD and PAC cassette integration are shown

To confirm the loss of the target genes in the knockout cell lines, the genomic DNA (gDNA) of each strain was isolated 10 days after transfection, and it was used as a template for a set of PCRs, as described in Fig. 1. The forward primer anneals between position 121 and 210 in the upstream intergenic region of the target genes, while the reverse primer anneals between position 223 and 428 in the internal region of the coding region. Thus, the annealing of the reverse primer varies according to each specific confirmation condition. The PCRs were performed using GoTaq^®^ Flexi DNA Polymerase (Promega), and the primers used are described in Supplementary Table S1. The reactions included GoTaq^®^ Flexi DNA Polymerase (5U), dNTPs (10 mM), forward primer (2 μM), reverse primer (2 μM) and MgCl_2_ (25 mM) in a final volume of 25 µl. The PCR protocol consisted of an initial denaturation step at 95 °C for 3 min, followed by 35 cycles of denaturation at 95 °C for 30 s, annealing at 60 °C for 30 s, extension at 72 °C for 1 min and 30 s and a final elongation step at 72 °C for 5 min. Afterwards, the amplicons were confirmed by electrophoresis on 1% agarose gel.

### Growth curves and cell differentiation

To evaluate the growth phenotype resulting from the deletion of the target genes in each modified strain, growth curve experiments were performed. Cultures of promastigotes from mutant cell lines and the control (*L. mexicana* T7/Cas9), which reached the stationary phase, were then diluted to an initial concentration of 1 × 10^5^ cells/ml in M199 medium with a pH of 7.4, supplemented with 10% FBS. These cultures were then incubated at 28 °C, and cell counts were done using a Neubauer chamber every 24 h, for a total of 96 h. All growth curves were performed in triplicate and the results plotted in graphs as mean ± SD (standard deviation) using GraphPad Prism version 6.

For metacyclogenesis, cultures of procyclic promastigotes at 1.5 × 10^5^ cells/ml were incubated in Grace’s medium, pH 5.5, supplemented with 4.62 mM of NaHCO_3_ for 7 days, followed by purification with 10 to 100% Percoll gradient, as described in [26]. The total number of cells was counted before the purification process, and only the metacyclics were counted after the gradient to calculate the metacyclogenesis ratio. The experiment was done with a minimum of four replicates.

For amastigogenesis assay, 5 × 10^6^ cells/ml of procyclic cultures were initially incubated at 28ºC in modified M199 (M199Ax), pH 5.2, supplemented with 2.5 μg/ml hemin, 0.1 mM adenine, 2.5 mg/ml d-glucose, 5 mg/ml peptone (trypticase), 40 mM sodium succinate (at pH 4.5), 2 mM l-glutamine, 20% FBS, 100 μg/ml penicillin and 100 μg/ml streptomycin for 1 day. The flasks were then transferred to storage at 33 ºC for the following 4 days. During the differentiation process, 1 × 10^7^ cells were collected daily for microscopy imaging. For this, the samples were washed three times with 1× PBS, incubated for 10 min in poly-l-lysine-coated slides, rewashed three times and fixed with 4% paraformaldehyde for 15 min. They were stained with Hoechst 33342 (1:1000) for 10 min and analyzed by fluorescence microscopy. Approximately 200 cells were counted per day to assess the differentiation rate. Two independent experiments were conducted, each in duplicate.

### mRNA sequencing

Total RNA was extracted from the mutant cell lines in triplicate and from the two controls, T7/Cas9 and WT, in quadruplicate. This extraction was performed using the RNeasy Mini Kit by Qiagen, following the manufacturer's instructions, with subsequent treatment involving DNAse I (Promega). To ensure the quality, integrity and concentration of the RNA, the samples were analyzed with the Qubit^™^ RNA IQ Assay Kit in Qubit 4 Fluorometer (Thermo Fisher Scientific) and agarose gel electrophoresis. To prepare paired-end sequencing libraries from the total RNA, we utilized the Stranded mRNA kit Prep by Illumina, following standard procedures. Subsequently, the libraries were sequenced on the Illumina NovaSeq™6000 sequencer using the 100-cycle sp v1.5 kit by Illumina.

### mRNA sequencing data analysis

The quality of the sequenced reads was checked using the FastQC tool. Each library was then mapped against the reference genome in FASTA format together with functional annotation in GFF format of *L. mexicana* strain MHOM/GT/2001/U1103 (downloaded from TriTrypdDB—https://tritrypdb.org/ release 56 from February 10, 2022) using STAR software [27], which was also used to produce the sequencing read count report considering the CDS region for each gene in the genome. The gene count data were loaded into the R environment and structured into a matrix, which was transformed to the log2 scale using the *rlog* transformation function of the DESeq2 package. This matrix was used as input for the *prcomp* function to perform principal component analysis (PCA). The R package DESeq2 version 1.22.1[28] was used to perform the global analysis of relative differential expression, considering only the genes represented by at least five reads for all replicates of at least one condition (control or modified strain). In addition, DESeq2 applies a negative binomial distribution to model the read counts, and we used the default hypothesis test of the package called Wald Test. The expression ratio (fold change, FC) of the genes between the control and the modified strain was obtained using a symmetrical scale transforming this ratio by the logarithmic function with base 2 (log2 fold change). Genes that exhibited an absolute log2 fold change equal ≥ 1 and false discovery rate (FDR) corrected *P*-values≤ 0.05 were considered differentially expressed genes (DEGs) and selected for further analyses. In addition, the correlation between corrected *P*-values and log2 fold change was also visualized using Volcano plots generated using the R package EnhancedVolcano version 1.18.0. (https://bioconductor.org/about/).

To functionally analyze the global impact of differentially expressed genes, we chose to perform a functional enrichment analysis through the String database (https://string-db.org/) using controlled vocabularies (ontologies) on Biological Process, Cellular Component and Molecular Function, all from the Gene Ontology (GO) consortium. This functional enrichment analysis employs a comprehensive approach by not only assessing the frequency of functional terms within a specified list of genes but also integrating expression rate metrics (log2 fold change), enabling the generation of a ranked list that reflects the combined influence of term occurrence and expression levels, thereby providing a more nuanced and informative insight into the biological significance of the genes under investigation.

### Motif enrichment analysis

The motif enrichment analysis was performed on the 3’ and 5’ UTR regions of the DEGs identified during the previous step. The genome used as reference for the expression analysis (MHOM/GT/2001/U1103) does not have the annotation for the UTR regions of the genes; therefore, the genome of *L. mexicana* MNYC/BZ/62/M379 strain (ref: 10.12688/wellcomeopenres.18575.2) was used. The gene IDs from both genomes were mapped comparing the gene sequences using BLAST alignment. Afterwards, the UTR sequences were downloaded from the cited study. The UTR sequences from both sides (3’and 5’) were used as input for a home-made pipeline written in NextFlow (https://www.nextflow.io) to orchestrate the motif analysis using the tools from MEME Suite (https://meme-suite.org/meme/index.html). Briefly, first a Markov model was built for each Fasta sequence file using the *fasta-get-markov* tool. Then, each Fasta sequencing file together with its respective model was used as input to MEME and STREME tools to identify the motifs. The motifs were statistically evaluated for enrichment in the dataset using the SEA, and finally the motifs were annotated using the Tomtom tool.

## Results

### Generation of TRMT61A and NSUN2 knockout cell lines

Based on the identified putative homologs encoding genes of TRMT61A and NSUN2 in *Leishmania* species [23], we attempted to generate knockout *L. mexicana* strains using the well-established CRISPR-Cas9 protocol [24]. After three independent attempts to delete both TRMT61A alleles, we observed the recovery of cells with only one allele deleted by replacement with donor DNA (-/+ , single knockout—SKO), suggesting that the presence of the two alleles might be essential for the viability of *L. mexicana.* On the other hand, we were able to obtain independent null mutants with both alleles replaced with donor DNA (-/-, double knockout, DKO) for NSUN2 genes. The TRMT61A and NSUN2 knockout cell lines were confirmed using conventional PCR reactions with locus-specific primers (Fig. 1).

### Cell differentiation of *Leishmania mexicana* is affected by TRMT61A and NSUN2 gene knockouts

**Fig. 2:**
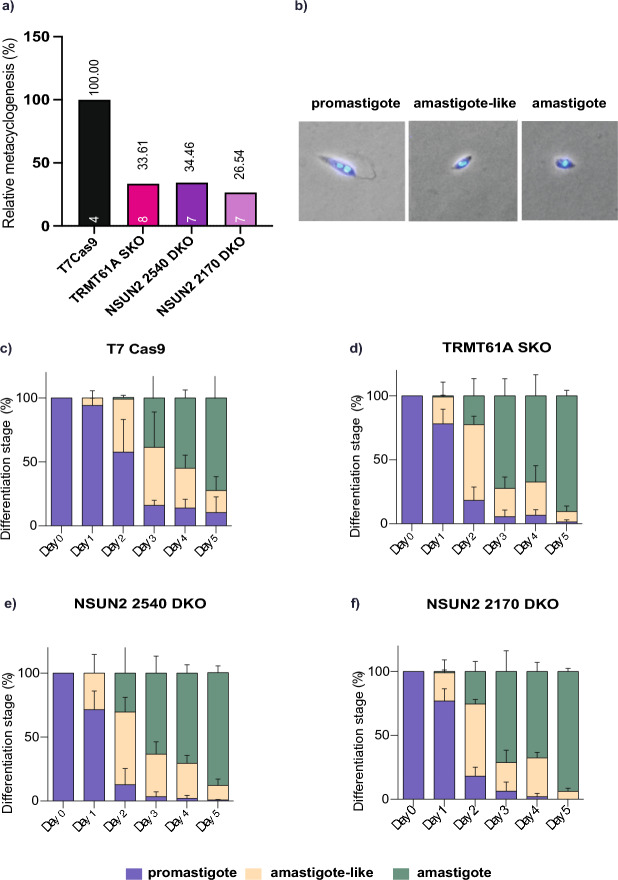
Cell growth and differentiation of TRMT61A and NSUN2 knockout cell lines. **a** Response of the three cell lines to in vitro metacyclogenesis, when the parasites were counted each 24 h, before and after Percoll gradient purification, to determine the percentage of metacyclic promastigotes relative to the control T7/Cas9. The number of replicates is represented within each bar. Statistical comparisons were made by one-way ANOVA followed by Dunnett's multiple comparisons test. **b** Representative images of each cell stage during amastigogenesis. Hoescht 33342 was used to stain the nucleus and kinetoplast. **c**–**f** Axenic amastigote differentiation and multiplication of control T7/Cas9 (**c**), TRMT61A SKO (**d**), NSUN2 2540 DKO (**e**) and NSUN2 2170 DKO (**f**). Logarithmic promastigotes were submitted to the axenic amastigote differentiation protocol, and the multiplication rate was monitored daily. Statistical comparisons were made by two-way ANOVA, followed by Tukey multiple comparisons test

To investigate any deleterious effect from the deletion of the target genes, we evaluated the TRMT61A and NSUN2 mutants during growth curves of the promastigote parasite stage compared to T7/Cas9 parental cell line. No apparent changes in morphology or decreases in growth rates were observed in any of the mutant cell lines (Additional file 2: Supplementary Fig. S1). Based on the results of PCR confirmation and growth curves, one clone of each mutant cell line was selected for further evaluation (clone 1, C1). The effect of deletions was then investigated on the differentiation process of *L. mexicana* (Fig. 2).

Initially, the metacyclic transformation capacity of mutant cell lines was observed and quantified compared to parental T7/Cas9. Using a differentiation rate of 100% for T7/Cas9 as a reference, the mutants exhibited average values < 35%. While the TRMT61A mutant showed a rate of 33.6%, NSUN2 2170 and 2540 cell lines presented only 26.5 and 34.6%, respectively. These findings indicate that, although the absence of TRMT61A and NSUN2 genes does not affect the normal growth of the procyclics, it can reduce the differentiation rate into metacyclic forms.

The differentiation from promastigote to axenic amastigote was also evaluated, using the following criteria of morphological changes: procyclic promastigote—elongated cell body with a long flagellum on the anterior portion and a clear distinction between the kinetoplast and nuclei; amastigote-like—oval cell body with nuclei and kinetoplast close together at the center and a small flagellum noticeable; amastigote—small oval cell body with no visible flagellum and no distinction between nuclei and kinetoplast.

Based on this, T7/Cas9 exhibited a promastigote proportion of 57.7% by day 2, whereas the highest proportion among the mutant lineages was only 18%. In the following days, the proportion of amastigotes was higher in all three mutants, which, by day 5, had approximately 90% of axenic amastigotes, while the control had a differentiation of 72.4%. These results suggest that the mutants may undergo earlier and faster differentiation into amastigotes.

### Bulk RNA sequencing of TRMT61A and NSUN2 mutant cell lines

In the sequencing experiments, three replicates of each modified cell line, TRMT61A SKO, NSUN2 2170 DKO and NSUN2 2540 DKO, and four of the controls, which were WT and T7/Cas9, were used, and a total of 453,937,475 reads were generated. They were subsequently mapped against the *L. mexicana* reference genome. We observed that on average the bases presented *phred* scores ≥ 30 (Table 1). Therefore, the removal of low-quality reads was not necessary. The number of reads between replicates uniquely mapped to the genome was generally uniform and around 70%. Then, the data were used for differential gene expression analysis using the DESeq2 package in the R programming environment. First, a gene expression analysis was performed between the T7/Cas9 and WT strain, and no significant difference was observed between the two strains (Additional file 3: Supplementary Table S2). Therefore, T7/Cas9 was chosen to continue as a control for comparison with the mutant strains. A principal component analysis (PCA) was also performed. In this step, transcriptomic sequencing data from the mutant strains were compared with data from the T7/Cas9 strain (control). Overall, two distinct groups of samples were observed for each mutant strain compared to the control (Additional file 4: Supplementary Fig. S2).

**Table 1:** Information regarding the quality of sequencing and library data

Sample	Total of reads	Total unambiguous mapping	%	Total multiple mapping	%	Total unmapped reads %	Average number of reads (millions)	Base percentage with Phred ≥ 30	
								R1 (%)	R2 (%)
WT	126,246,514	92,700,900	73.43	27,365,528	21.68	4.90	31	94	91
T7/Cas9	123,943,367	89,810,176	72.46	26,075,791	21.04	6.50	30	93	92
TRMT61A	71,825,675	50,821,792	70.76	12,848,319	17.89	11.35	23	92	92
NSUN2 2170	83,220,589	61,654,937	74.09	15,413,772	18.52	7.39	27	94	92
NSUN2 2540	86,342,687	60,703,662	70.31	16,429,509	19.03	10.67	28	93	91

### Global impact on gene expression due to the deletion of TRMT61A and NSUN2 genes

**Fig. 3:**
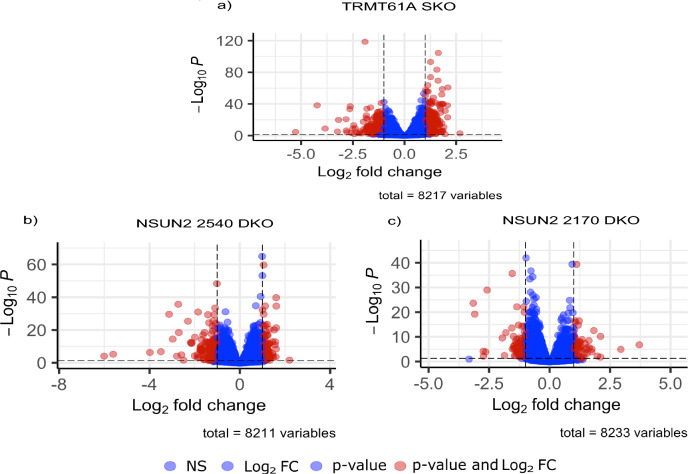
Volcano plots of differentially expressed genes in the TRMT61A and NSUN2 mutants compared to parental cell line. The graphs show the fold change (x-axis) versus corrected *p*-value (y-axis) of the analyzed genes. The significance (adjusted *p*-value) and fold change are converted to − Log10 (*p*-value) and Log2 (fold change), respectively. The vertical and horizontal dotted lines show the cutoff of log2 fold change = ± 1 and – log10 correct *p* = 0.05, respectively. **a** For TRMT61A SKO, there were 239 upregulated genes and 178 downregulated. **b** For NSUN2 2540 DKO, 91 were upregulated and 130 downregulated. **c** For NSUN2 2170 DKO, 54 were upregulated and 55 downregulated

Differential expression analysis of TRMT61A mutant compared to T7/Cas9 confirmed the generation of single-knockout parasites as a significant downregulation of TRMT61A transcripts, with a log_2_ fold change of 1.06, corresponding to a twofold reduction. Moreover, we found that from the 8217 genes evaluated, 417 exhibited significant differences in expression, characterized by absolute log_2_ fold change values ≥ 1 and corrected *p*-values ≤ 0.05 (Fig. 3a and Additional file 5: Supplementary Table S3). Among these differentially expressed genes (DEGs), 239 were upregulated, while 178 were downregulated and assigned to different cellular processes, including genes related to the biological context of RNA metabolism.

The comprehensive transcriptomic sequencing analysis of NSUN2 null mutants revealed a striking 64-fold reduction (log_2_ fold change = −6) for NSUN2 2540 DKO transcripts and eightfold difference (log_2_ fold change = −3) for NSUN2 2170 DKO compared to T7/Cas9 parental cell line. This result, combined with the analysis of the RNA-seq read coverage at the TRMT61A and NSUN2 *loci* in T7/Cas9 and mutant lines (Additional file 6: Supplementary Fig. S3), confirms the successful deletion of each gene in their specific cell line. The differential gene expression analysis for lineages with the deleted genes for NSUN2 2540 and NSUN2 2170 involved 8211 and 8233 genes, respectively. The cell line with the NSUN2 2540 deletion displayed 221 DEGs, comprising 130 downregulated and 91 upregulated (Fig. 3b and Additional file 7: Supplementary Table S4). Conversely, the lineage lacking the NSUN2 2170 gene showed 109 DEGs, with 55 downregulated and 54 upregulated (Fig. 3c and Additional file 8: Supplementary Table S5).

**Fig. 4:**
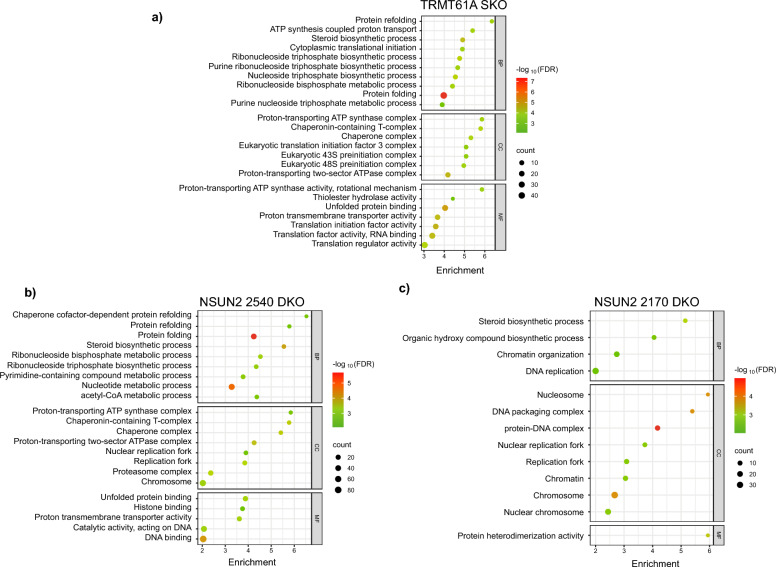
Gene enrichment analyses of differentially expressed genes (DEGs) found in TRMT61A and in both NSUN2 mutants compared to parental cells. The analyses were done based on the Gene Ontology Consortium for Biological Process (BP), Cellular Component (CC) and Molecular Function (MF) for **a** TRMT61A SKO, **b** NSUN2 2540 DKO and **c** NSUN2 2170 DKO. The size of each circle in the graphs represents the number of differentially expressed genes associated with a given functional term. The color scale represents the *p*-value converted to a base 10 negative logarithmic scale, thus illustrating the statistical significance of the enrichment of a given functional term. The plots were generated using SRplot (bioinformatics.com.cn/srplot)

To analyze the functional impacts of the differentially expressed genes, we performed gene enrichment analysis based on Gene Ontology (GO) terms for Biological Process (BP), Cellular Component (CC) and Molecular Function (MF) (Fig. 4). Looking at the functional enrichment results for the TRMT61A mutant, among the BP terms with the highest scores, we found protein folding and refolding as well as terms that suggest a possible disturbance in pathways involved in metabolism of nucleotides (Fig. 4a). Furthermore, important structures involved in the metabolism of mRNAs were affected, such as the eIF3 translation initiation complex, the eukaryotic 43S translation pre-initiation complex and chaperone complexes, as observed in the CC group. Finally, several MF terms related to translation were also detected.

Comparing the two mutant lineages of the NSUN2 genes, there were more pronounced changes in the mutant for NSUN2 2540, particularly with terms of BP related to protein folding and refolding and nucleotide metabolism (Fig. 4b). Energetic metabolism and chaperone complexes were identified in cellular components as well as terms related to DNA replication. These last two functions were also identified in the terms of MF. For the other NSUN2 mutant (2170), the profile for GO enrichment was different, and a small number of identified terms reflected the reduced number of DEGs observed in this cell line (Fig. 4c). Regarding BP, the changes occurred in biosynthetic components, while for CC, there were genes related to the terms involving DNA and DNA-protein complexes as well as nucleosomes. For MF, the enriched genes were related to only one term, which was protein heterodimerization activity.

### Overview of differentially expressed transcripts in TRMT61A single-knockout cell line

Following the analysis of the general transcriptomic profile after deletion of the genes of interest, we subsequently conducted a more detailed analysis of the transcripts that were most affected within each cell line. The top 30 most differentially expressed genes in the TRMT61A SKO mutant are shown in Table 2. For negatively regulated genes, the TOP1 mRNA is from a hypothetical protein (LmxM.01.0830), with a 27.8-fold decrease in expression. Other highly impacted downregulated genes were LmxM.24.1840, a lysophospholipase; LmxM.32.1760, an OTT_1508-like deaminase, with a 10.5-fold difference in expression; LmxM.24.1850, a hypothetical predicted multi-pass transmembrane protein, and LmxM.05.1215, a surface antigen-like protein, both with an eightfold difference in expression. Many mRNAs encoding hypothetical proteins were noticed, and LmxM.08_29.1090, encoding ribosomal protein L1a, showed a fourfold difference in expression.

**Table 2:** List of the 30 most differentially expressed genes in *Leishmania mexicana* promastigotes with a single knockout of the TRMT61A-encoding gene

TRMT61A SKO—downregulated			TRMT61A SKO—upregulated		
TriTrypDB accession	Annotation	Fold change	TriTrypDB accession	Annotation	Fold change
LmxM.01.0830	Hypothetical protein	27.80	LmxM.26.0640	10 kDa heat shock protein, putative	4.42
LmxM.24.1840	Lysophospholipase, putative	17.87	LmxM.32.2390	Heat shock protein, putative	4.36
LmxM.32.1760	OTT_1508-like deaminase, putative	10.54	LmxM.29.0730	Co-chaperone GrpE, putative	4.31
LmxM.24.1850	Hypothetical predicted multi-pass transmembrane protein	8.00	LmxM.09.0930	Calmodulin, putative	3.87
LmxM.05.1215	Surface antigen-like protein	7.92	LmxM.32.0316	Heat shock protein 83–1	3.85
LmxM.33.1600	Hypothetical protein	7.75	LmxM.04.0310	Beta-fructofuranosidase, putative	3.64
LmxM.24.1830	Hypothetical protein, conserved	7.00	LmxM.24.0630	ATPase subunit 9, putative	3.50
LmxM.04.0130	Hypothetical protein	6.90	LmxM.29.3090	RNA-binding protein 42 (RNA-binding motif protein 42), putative	3.45
LmxM.16.0570	Histone H3	6.64	LmxM.03.0600	Arginine N-methyltransferase, putative	3.36
LmxM.05.0480	Monocarboxylate transporter-like protein	6.25	LmxM.18.0740	Elongation factor Tu, mitochondrial, putative	3.35
LmxM.18.1350	Hypothetical protein, conserved	6.03	LmxM.36.2030	Chaperonin HSP60, mitochondrial precursor	3.33
LmxM.08.1171	Hypothetical protein	5.90	LmxM.18.0020	Diphosphomevalonate decarboxylase, putative	3.26
LmxM.33.1580	Hypothetical protein	5.70	LmxM.36.2020	Chaperonin HSP60, mitochondrial precursor	3.26
LmxM.26.1780	Hypothetical protein, unknown function	5.23	LmxM.25.2140	Succinyl-CoA synthetase alpha subunit, putative	3.26
LmxM.12.1090	Promastigote surface antigen protein PSA	4.79	LmxM.19.1315	Hypothetical protein, conserved	3.14
LmxM.33.0070	Ascorbate peroxidase, putative	4.62	LmxM.29.1610	Ferric reductase transmembrane protein, putative	3.08
LmxM.08_29.1090	Ribosomal protein L1a, putative	4.40	LmxM.28.2080	DEAD-box ATP-dependent RNA helicase, mitochondrial	3.06
LmxM.04.0190	Surface antigen-like protein	3.99	LmxM.30.2600	Calreticulin, putative	3.05
LmxM.34.5000	PSP1 C-terminal conserved region, putative	3.75	LmxM.19.1485	Hypothetical protein, conserved	2.98
LmxM.28.2480	Hypothetical protein, conserved	3.67	LmxM.03.0180	Zinc-finger of acetyl-transferase ESCO, putative	2.94
LmxM.08.1060	Cathepsin L-like protease, putative	3.60	LmxM.32.3240	H1 histone-like protein	2.87
LmxM.23.1665	PAP2 superfamily, putative	3.44	LmxM.18.0150	Serine/threonine protein phosphatase type 5, putative	2.86
LmxM.23.1155	Hypothetical predicted multi-pass transmembrane protein	3.41	LmxM.26.2305	Hypothetical protein, conserved	2.77
LmxM.19.0870	ATG8/AUT7/APG8/PAZ2, putative	3.40	LmxM.29.2470	Heat shock 70-related protein 1, mitochondrial precursor, putative	2.70
LmxM.33.1560a	Hypothetical protein	3.38	LmxM.32.1140	Hypothetical protein, conserved	2.68
LmxM.30.0450b	Hypothetical protein	3.33	LmxM.29.2490	Heat shock 70-related protein 1, mitochondrial precursor, putative	2.65
LmxM.08_29.0251	Thymine-7-hydroxylase, putative	3.32	LmxM.32.1630	Cyclophilin, putative	2.64
LmxM.24.1860	Hypothetical protein, conserved	3.20	LmxM.07.0100	Pitrilysin-like metalloprotease, metallo-peptidase, Clan ME, Family M16C	2.64
LmxM.33.1590	Tuzin-like protein	3.20	LmxM.19.0210	ADP/ATP translocase 1, putative, ADP,ATP carrier protein 1, mitochondrial precursor, putative	2.60
LmxM.05.0900	Surface antigen-like protein	3.12	LmxM.07.0640	Eukaryotic translation initiation factor 3 subunit h	2.55

Analysis of the most upregulated mRNAs revealed some genes associated with the heat shock response, including three with a high fold change (≥ 3.8-fold), classified as heat shock proteins (LmxM.26.0640, LmxM.32.2390 and LmxM.32.0316). Other genes annotated as coding for HSP60 chaperonins and mitochondrial heat shock 70-related protein 1 were also found. Among the top-most positively regulated DEGs, we identified genes related to mRNA metabolism and the translation process, such as the RNA binding protein 42 (RBP42) (LmxM.24.0630) and the subunit H of the eukaryotic eIF3 translation initiation complex (LmxM.07.0640) with a 2.5-fold increase, while other subunits of eIF3 complex (A, B, C, D and I) were also upregulated, all showing a twofold difference in expression (Supplementary Table S3).

The results show that the deletion of TRMT61A gene, although partial, affected many transcripts of diverse categories, some including components of the translation initiation machinery and nucleotide metabolism. This evidence suggests a possible effect of TRMT61A enzyme in processes involved in gene expression regulation in *L. mexicana*.

### Overview of transcripts differentially expressed in NSUN2 mutants

Within the NSUN2 2540 DKO mutant, the TOP1 downregulated mRNA was its respective transcript. The profile for most downregulated genes included the hypothetical protein (LmxM.01.0830), with a 31.4-fold decrease in expression, a ribosomal protein L1a (LmxM.08_29.1090) and the OTT_1508-like deaminase (LmxM.32.1760). The list also includes genes for autophagy-related proteins (ATG8/AUT7/APG8/PAZ2) and many hypothetical and surface antigen-like proteins (Table 3).

**Table 3:** List of the 30 most differentially expressed genes in *Leishmania mexicana* promastigotes with a double knockout of the NSUN2 2540-encoding gene

NSUN2 2540 DKO—downregulated			NSUN2 2540 DKO—upregulated		
TriTrypDB accession	Annotation	Fold change	TriTrypDB accession	Annotation	Fold change
LmxM.08_29.2540	NOL1/NOP2/sun family, putative	36.14	LmxM.26.0640	10 kDa heat shock protein, putative	2.61
LmxM.01.0830	Hypothetical protein	31.45	LmxM.09.0930	Calmodulin, putative	2.60
LmxM.08_29.1090	Ribosomal protein L1a, putative	15.86	LmxM.18.0020	Diphosphomevalonate decarboxylase, putative	2.60
LmxM.32.1760	OTT_1508-like deaminase, putative	12.21	LmxM.08_29.1720	Histone H2A, putative	2.56
LmxM.24.1840	Lysophospholipase, putative	9.82	LmxM.29.1610	Ferric reductase transmembrane protein, putative	2.54
LmxM.04.0130	Hypothetical protein	7.41	LmxM.32.2390	Heat shock protein, putative	2.46
LmxM.24.1850	Hypothetical predicted multi-pass transmembrane protein	7.30	LmxM.32.3240	H1 histone-like protein	2.40
LmxM.05.1215	Surface antigen-like protein	6.69	LmxM.29.0730	Co-chaperone GrpE, putative	2.32
LmxM.24.1830	Hypothetical protein, conserved	5.27	LmxM.36.2030	Chaperonin HSP60, mitochondrial precursor	2.20
LmxM.14.0470	UDP-glucoronosyl and UDP-glucosyl transferase, putative	4.73	LmxM.36.0020	Histone H4	2.13
LmxM.18.1350	Hypothetical protein, conserved	4.59	LmxM.29.3090	RNA-binding protein 42 (RNA-binding motif protein 42), putative	2.09
LmxM.08_29.0251	Thymine-7-hydroxylase, putative	4.52	LmxM.28.2080	DEAD-box ATP-dependent RNA helicase, mitochondrial	2.07
LmxM.33.0070	Ascorbate peroxidase, putative	4.00	LmxM.25.1180	ATP synthase subunit beta, mitochondrial, putative	1.83
LmxM.04.0190	Surface antigen-like protein	3.95	LmxM.25.2140	Succinyl-CoA synthetase alpha subunit, putative	1.78
LmxM.09.0150	ATG8/AUT7/APG8/PAZ2, putative	3.51	LmxM.34.4470	Co-chaperone protein P23	1.75
LmxM.26.1780	Hypothetical protein, unknown function	3.50	LmxM.13.0690	PSP1 C-terminal conserved region, putative	1.75
LmxM.30.0450b	Hypothetical protein	3.41	LmxM.30.2600	Calreticulin, putative	1.73
LmxM.33.1600	Hypothetical protein	3.31	LmxM.18.0150	Serine/threonine protein phosphatase type 5, putative	1.67
LmxM.33.1580	Hypothetical protein	3.09	LmxM.21.1090	T-complex protein 1, delta subunit, putative	1.64
LmxM.14.0720	Hypothetical protein	3.04	LmxM.23.0760	Mitochondrial RNA binding protein, putative	1.58
LmxM.09.0180	ATG8/AUT7/APG8/PAZ2, putative	3.03	LmxM.36.5845	Kinetoplast-associated protein, putative	1.58
LmxM.19.0870	ATG8/AUT7/APG8/PAZ2, putative	2.93	LmxM.25.1170	ATP synthase subunit beta, mitochondrial, putative	1.58
LmxM.04.0210	Surface antigen-like protein	2.90	LmxM.24.2110	3-hydroxy-3-methylglutaryl-CoA synthase, putative	1.58
LmxM.05.0900	Surface antigen-like protein	2.89	LmxM.32.1630	Cyclophilin, putative	1.58
LmxM.04.0180	Surface antigen-like protein	2.84	LmxM.25.2450	Histone H4	1.57
LmxM.33.1560a	Hypothetical protein	2.81	LmxM.13.1620	Squalene monooxygenase-like protein	1.54
LmxM.33.1720	Hypothetical protein	2.66	LmxM.29.2490	Heat shock 70-related protein 1, mitochondrial precursor, putative	1.53
LmxM.23.1665	PAP2 superfamily, putative	2.65	LmxM.24.0630	ATPase subunit 9, putative	1.53
LmxM.09.0690	Hypothetical protein, conserved	2.63	LmxM.04.0310	Beta-fructofuranosidase, putative	1.52
LmxM.12.1090	Promastigote surface antigen protein PSA	2.53	LmxM.06.0860	Dihydrofolate reductase-thymidylate synthase	1.52

For NSUN2 2170 DKO, the list of the top downregulated genes includes many of those coding for hypothetical proteins, surface antigen-like protein and tuzin-like proteins (LmxM.33.1590, LmxM.33.1570, LmxM.33.1610, LmxM.33.1810, LmxM.34.1830 and LmxM.34.1830a), which seemed to be part of a cluster (Table 4). Within the list of top positively enriched hits, two genes for the glycoprotein GP63 (LmxM.10.0390 and LmxM.10.0470) were present. Different histone-coding mRNAs were also found and many transcripts of metabolic enzymes, some related to lipid metabolism (Supplementary Table S5).

**Table 4:** List of the 30 most differentially expressed genes in *Leishmania mexicana* promastigotes with a double knockout of the NSUN2 2170-encoding gene

NSUN2 2170 DKO—downregulated			NSUN2 2170 DKO—upregulated		
TriTrypDB accession	Annotation	Fold change	TriTrypDB accession	Annotation	Fold change
LmxM.24.1840	Lysophospholipase, putative	9.96	LmxM.04.0310	Beta-fructofuranosidase, putative	4.43
LmxM.24.1850	Hypothetical predicted multi-pass transmembrane protein	9.58	LmxM.10.0390	GP63, leishmanolysin	3.34
LmxM.01.0830	Hypothetical protein	7.53	LmxM.36.0020	Histone H4	3.06
LmxM.08.1171	Hypothetical protein	7.48	LmxM.25.2450	Histone H4	2.69
LmxM.08_29.1090	Ribosomal protein L1a, putative	6.99	LmxM.08_29.1720	Histone H2A, putative	2.64
LmxM.24.1830	Hypothetical protein, conserved	6.67	LmxM.29.1610	Ferric reductase transmembrane protein, putative	2.39
LmxM.33.1590	Tuzin-like protein	3.82	LmxM.04.0320	Hypothetical protein	2.34
LmxM.05.1215	Surface antigen-like protein	3.48	LmxM.30.3180	Histone H4	2.25
LmxM.05.0900	Surface antigen-like protein	3.26	LmxM.24.2110	3-hydroxy-3-methylglutaryl-CoA synthase, putative	2.08
LmxM.30.0460e	Hypothetical protein	2.43	LmxM.03.0180	Zinc-finger of acetyl-transferase ESCO, putative	1.79
LmxM.28.1600	Helicase-like protein	2.39	LmxM.18.0150	Serine/threonine protein phosphatase type 5, putative	1.64
LmxM.08.1060	Cathepsin L-like protease, putative	2.38	LmxM.30.1190	Cytochrome b5-like Heme/Steroid binding domain containing protein, putative	1.62
LmxM.33.1570	Tuzin-like protein	2.23	LmxM.19.0920	Pteridine transporter, putative	1.52
LmxM.18.1350	Hypothetical protein, conserved	2.20	LmxM.10.0470	GP63, leishmanolysin	1.51
LmxM.19.0870	ATG8/AUT7/APG8/PAZ2, putative	2.19	LmxM.18.0020	Diphosphomevalonate decarboxylase, putative	1.49
LmxM.05.0241	Viscerotropic leishmaniasis antigen, putative	2.18	LmxM.36.0010	Phosphoglycan beta 1,3 galactosyltransferase 4	1.49
LmxM.33.0070	Ascorbate peroxidase, putative	2.11	LmxM.21.0740	ATPase subunit 9, putative	1.48
LmxM.33.1610	Tuzin-like protein	2.05	LmxM.09.0930	Calmodulin, putative	1.47
LmxM.04.0390	Hypothetical protein	1.99	LmxM.34.1310	Histone H4	1.43
LmxM.33.1600	Hypothetical protein	1.94	LmxM.32.1330	Glutamine aminotransferase, putative	1.40
LmxM.21.0520	Nucleoporin NUP41	1.87	LmxM.23.0730	RNA-binding protein, putative	1.40
LmxM.29.2090	Alcohol dehydrogenase, putative	1.85	LmxM.24.2230	Heme Response-1 protein, putative	1.39
LmxM.33.1730	Hypothetical protein	1.83	LmxM.34.1890	60S ribosomal protein L5, putative	1.39
LmxM.33.1810	Tuzin-like protein	1.73	LmxM.13.1620	Squalene monooxygenase-like protein	1.29
LmxM.34.1830a	Tuzin-like protein	1.68	LmxM.32.3240	H1 histone-like protein	1.29
LmxM.33.1830	Tuzin-like protein	1.65	LmxM.08_29.2390	Kinesin, putative	1.26
LmxM.08_29.0251	Thymine-7-hydroxylase, putative	1.64	LmxM.30.0800	Hypothetical protein, conserved	1.25
LmxM.16.1140	WW domain containing protein, putative	1.56	LmxM.32.1590	3-ketoacyl-CoA reductase, putative	1.25
LmxM.14.0720	Hypothetical protein	1.55	LmxM.25.2460	Phosphoglycan beta 1,3 galactosyltransferase 6	1.18
LmxM.30.0460a	Hypothetical protein	1.54	LmxM.08_29.0850	High mobility group protein TDP1	1.13

### Comparison of the differentially expressed genes in TRMT61A and NSUN2 mutants

**Fig. 5:**
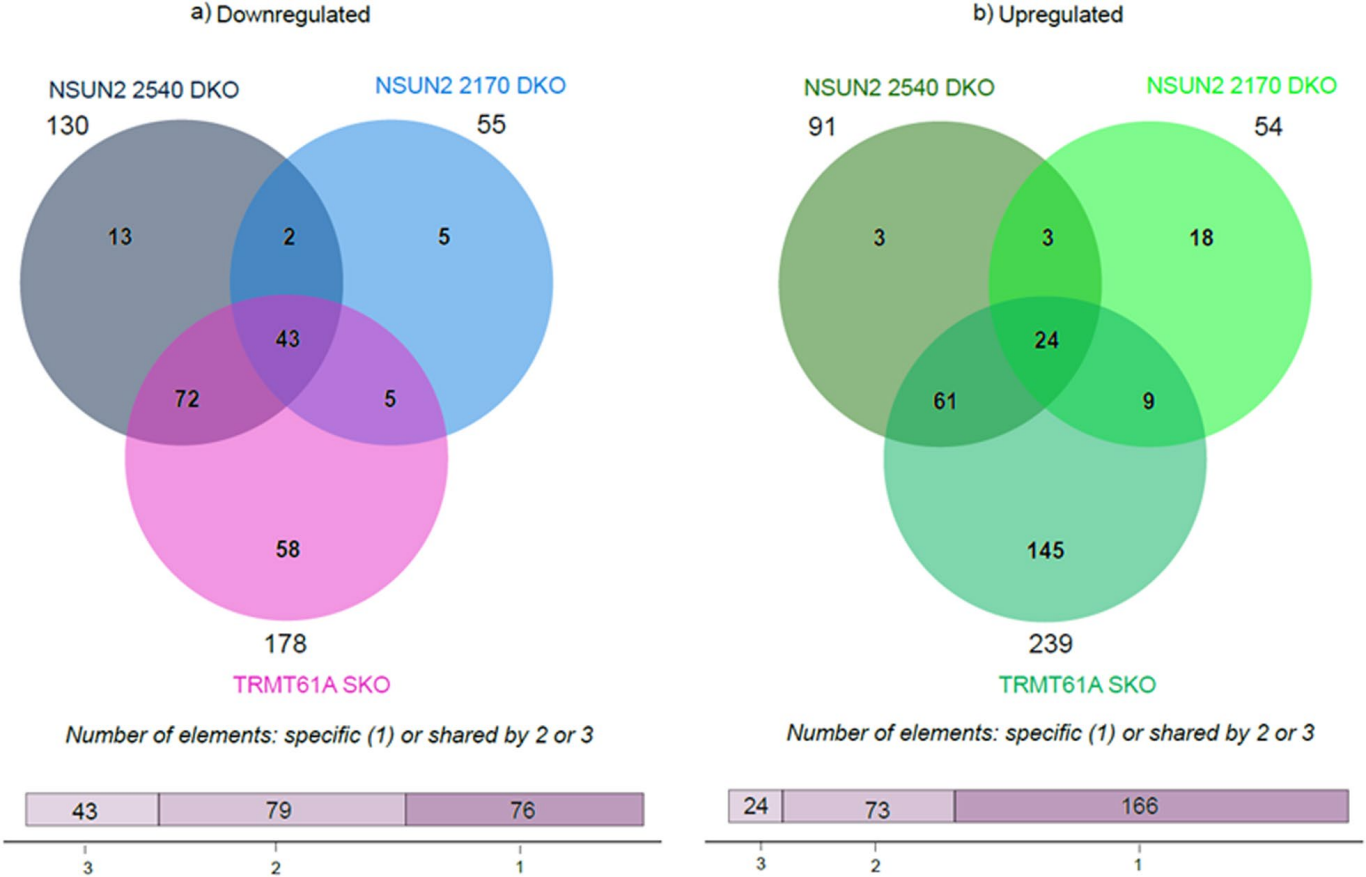
Comparative analysis of differentially expressed genes among the three different mutant cell lines. Venn diagram showing the intersection among the transcripts present at TRMT61A SKO and DKO of the orthologs NSUN2 2450 and NSUN2 2170, where **a** is for downregulated mRNAs and **b** for upregulated. The plots were generated using SRplot (bioinformatics.com.cn/srplot)

Many transcripts with a significant impact on expression levels were identical or similar between the different mutant strains. When the results from the three mutant cell lines were compared, a greater impact was observed on the negatively regulated genes which exhibited higher fold changes (27.8-, 31.5- and 10-fold for TRMT61A, 2540 and 2170, respectively) compared to positively regulated genes (4.4-, 2.6- and 3.4-fold, respectively). A Venn diagram illustrates the intersection among the different mutants, identifying 43 common transcripts in the list of downregulated DEGs, whereas 24 upregulated DEGs are common to all three strains (Fig. 5). Notably, in the lists of the top 30 most downregulated, 12 genes were present in all 3 cell lines. Fewer transcripts were common when the lists of the TOP30 upregulated ones were compared, with only six being present in all three cell lines.

The comparison also highlights that most of the DEGs identified in the NSUN2 2540 DKO strain are also subject to some form of regulation in the remaining cell lines, with many corresponding to the TRMT61A mutant. Of the 130 downregulated genes, 115 were also present in the TRMT61A SKO, whereas only 45 were identified in the NSUN2 2170 DKO. Similarly, for the upregulated genes, 85 of the 91 are common between NSUN2 2540 DKO and TRMT61A SKO, while the NSUN2 orthologs share 27 common transcripts. There is also an intersection between TRMT61A SKO and NSUN2 2170 DKO, with 48 downregulated genes and 33 upregulated genes in common. This set of results suggests a potential interplay among the three methyltransferases orthologs.

### Motif discovery analysis

The observation that several of the genes with high impact on expression levels were identical or similar among the different mutant strains led us to seek potential common RNA motifs in the regulated transcripts. For this, each set of 5′ UTR and 3′ UTR sequences from positively or negatively differentially expressed genes in each strain were analyzed using tools from the MEME Suite.

Although motifs associated with classical readers of chemical modifications, such as YTH domain-containing proteins, YBX1 and ALYREF, were not clearly identified in the sequences of the RNA sets, the results revealed sequences recognized by multiple RBPs, including hnRNPs, PABP, PCBPs and RBMs, among others (Additional file 9: Supplementary Table S6). The highest enrichment values were observed for the hnRNPs (K and L) in motifs mostly present on 5’ UTRs of upregulated transcripts. Notably, no significant motifs were detected in the 5’ UTRs of downregulated mRNAs. A motif rich in cytosines and adenines (ACACACACAC) related to recognition by IGF2BPs (*Insulin-like growth factor 2 mRNA-binding proteins*) was found in the 3’UTR set of sequences from upregulated genes in the DKO cell line for the NSUN2 2540 gene. IGF2BPs have been reported as non-canonical methylation readers, with IGF2BP2 being a protein that preferentially binds to N6-methyladenosine (m6A)-containing mRNAs and increases their stability [29, 30].

## Discussion

Trypanosomatids, such as *Leishmania*, depend on efficient gene expression regulation to modulate the levels of transcripts that encode proteins that are necessary for cell multiplication and differentiation during host exchange. This regulation occurs mainly in the post-transcriptional stages, which includes the processing, stability, transport and translation of mRNAs [2]. In the context of post-transcriptional regulation, epitranscriptomic studies have enabled the identification of chemical nucleotide modifications, such as m1A and m5C, within mRNA structures. These modifications play key roles in post-transcriptional mechanisms, particularly in regulating mRNA stability and translation efficiency [8].

Cellular differentiation is one of the mechanisms through which chemical modifications have been studied, and these RNA modifications have been considered the main factors involved in the pluripotency of stem cells, embryonic development and progression of diseases such as cancer [31]. In humans, the deletion of METTL3, writer of m6A, interrupts cell renewal, and this observation reinforces the participation of chemical modifications in cell differentiation processes [32].

In this work, the deletion of one allele of the TRMT61A gene or both alleles of each NSUN2 ortholog did not affect the growth of *L. mexicana* promastigote forms. The absence of substantial impacts on the growth kinetics of the mutants, in the procyclic form, can be rationalized by the nature of a single knockout, as observed in the case of TRMT61A. In this scenario, the presence of an additional copy of the gene is likely to provide some level of compensation for the allele loss, thereby preserving essential functions in this stage. This concept is similarly applicable to NSUN2, even in the context of a double knockout, as *L. mexicana* features another NSUN2 gene with a conserved domain for methyltransferase activity, implying a redundancy that could mitigate the overall impact on growth kinetics despite the targeted disruptions. However, in both cases, these genes seem to impact the parasite growth and development in adverse conditions. Indeed, the gene knockouts could alter the parasite’s differentiation profile into amastigotes and reduce the metacyclogenesis rate by > 65%. In a similar approach, Maran et al. [26] evaluated the growth and differentiation of *L. mexicana* clones with a single knockout for the homolog of the enzyme N-acetyltransferase (NAT10), which is responsible for mRNA acetylation (ac4C). In the study, the authors also observed effects on parasite growth, although in a different pattern from that observed here. The knockout of NAT10 in *L. mexicana* resulted in slower multiplication of the procyclic stage compared to the T7/Cas9 control, without significantly affecting metacyclogenesis. This phenotype was associated with increased proliferation of axenic amastigotes [26].

In trypanosomatids, m6A is the best characterized mRNA modification. It is highly abundant throughout the transcriptome of *T. brucei*, where VSG transcripts are methylated with m6A in the poly A-tail, and the 16-mer motif (5'-TGATATATTTTAACAC-3') is the binding region of the possible writer of this modification. When this region is mutated, the m6A modification is lost, causing rapid deadenylation of the poly A tail and reduced stability of the VSG mRNA [21]. Our results of differential expression analysis showed a negative regulation of surface proteins in *L. mexicana*, indicating that the knockout of TRMT61A and NSUN2 writers might potentially reduce the formation of these modifications and consequently reduce the transcripts for surface proteins. For example, the promastigote PSA surface antigen-like protein and surface antigen-like protein annotated genes presented a sixfold difference downregulation in both TRMT61A and NSUN2 mutants. These surface proteins are expressed in cell differentiation as a response to environmental changes and are present in both amastigote and promastigote forms, but they are more expressed in promastigotes [33]. Therefore, this group of evidence suggests that modified transcripts may play a pivotal role in the expression of proteins essential for the developmental stages of *L. mexicana*.

It is known that, under stress or during cellular differentiation, RNA chemical modifications regulate mRNA stability and promote translation control [34, 35]. In *Plasmodium falciparum*, although no growth defect on parasite proliferation was observed, the knockout of Pf-DNMT2, a methyltransferase that adds m5C methylation to tRNA, caused the downregulation in a subset of proteins that shifts the parasite metabolism, influencing the response to cellular stress [36]. The chemical modification m5C also confers stability to transcripts associated with the gametogenesis process in *Plasmodium spp*., as mRNAs methylated with m5C have a longer half-life compared to transcripts without the modification, and a decrease in m5C levels negatively impacts the gametocyte formation [37]. In *T. brucei*, it was seen that chemical modifications may help stabilize protein-RNA interactions, as the presence of pseudouridines strengthens protein-RNA interactions, mostly at high temperatures [38].

Through enrichment analysis of the DEGs in the TRMT61A mutant, we identified transcripts related to terms such as 'protein folding ' and ‘protein refolding,” and the same terms appeared in the analysis of NSUN2 2540 DKO. Synonymous mutations, characterized by a single nucleotide exchange without altering the amino acid sequence, carry significant implications for protein folding dynamics. These observations indicate that alterations in highly frequent codons, leading to changes in codon usage, result in a deceleration of translation speed in regions previously characterized by faster translation. This, in turn, influences the folding process of proteins being translated [39]. The m1A modification is present in the mRNA structures, and it is involved in pairing or interacting with elements of the translation machinery [40]. Therefore, it is possible to have a similar mechanism in *L. mexicana*, where changes in translation speed, and consequent impact on protein folding, might occur because of the lack of an adequate level of mRNA modifications, suggesting a potential link between translation dynamics and maintenance modifications on mRNAs. Our global transcriptome analysis also revealed an increased frequency of terms related to nucleotide metabolism in both mutants. *Leishmania* relies on the nucleotide metabolism machinery for both de novo pyrimidine synthesis and purine recycling processes [41]. The salvage of purines involves the translocation of nucleotides facilitated by proteins responsible for transporting pre-formed purines [42]. Genes associated with the dihydrofolate reductase, adenosine kinase and other key proteins involved in both synthesis and purine recycling processes were identified in the gene list enriched with a twofold positive regulation for both mutants. Furthermore, enrichment terms connected with the formation of the translation initiation complex, including 43S and 48S pre-initiation complexes, are particularly evident in the TRMT61A SKO dataset. The genes annotated to these terms include eIF3 translation initiation and elongation factors, exhibiting a positive regulation of twofold difference. These findings might suggest a potential compensatory mechanism in response to the reduction of chemical modifications. This compensation might involve an upregulation in the expression of key elements within the protein synthesis and nucleotide metabolism machinery, aiming to sustain translation levels for transcripts affected in the mutants.

Although GO enrichment analyses for TRMT61A and NSUN2 2540 mutants highlighted similarities, surprisingly, the two orthologs of the NSUN2 methyltransferase did not show many terms in common. This may indicate that, despite presenting methyltransferase motifs, these proteins may perform related but not redundant functions. In the DEGs list for the NSUN2 2170 mutant, several transcripts of tuzin-like proteins with negative regulation are present. Tuzins are transmembrane proteins with unknown function but are frequently associated with amastins. In *Leishmania*, they have been evaluated as a vaccine candidate due to their antigenic properties [43, 44]. According to the functional enrichment analysis of the two different sets of DEGs for the NSUN2 paralogs, one can observe a potential functional divergence between the two genes. Paralog NSUN2 2540 has a set of functional terms indicating a participation of more dynamic cell processes, whereas the functional terms from the paralog NSUN2 2170 indicate its participation in processes related to the cell structure. However, which signal is targeted by these two different NSUN2 proteins is unclear.

The epitranscriptomic machinery usually recognizes specific sequences in the RNA to be modified [45]. Here, a comprehensive analysis was performed to identify potential sequence motifs enriched among differentially expressed mRNAs. Although no distinct motif could be unequivocally associated with either m1A or m5C modifications, sequences recognized by certain RNA-binding proteins (RBPs) were detected, which have been previously linked to RNA methylations and non-canonical ‘readers’ [29, 46].

Our work adds information about chemical modifications of RNA in *L. mexicana*, which to date have been little explored in trypanosomatids. We show evidence of differences in expression of some genes and the impact on biological processes because of TRMT61A and NSUN2 ortholog knockout; however, the complete sets of binding proteins and modification dynamics remain unknown in trypansomatids, showing the need for further studies to fill these gaps.

A major challenge to studying epitranscriptomes is still the limited availability of sensitive, high-throughput approaches to detect and analyze modified RNA molecules in a transcriptome-wide manner [47]. Integrating methodologies such as CLIP-seq for ‘reader’ proteins with modification-specific sequencing is important for advancing our understanding of the functional consequences of RNA modifications [48]. New technologies are arising to help circumvent this issue, such as nanopore sequencing, allowing direct sequencing on the RNA molecule without requiring additional steps to convert RNA to cDNA [49]. Thus, future studies applying technologies to detect the mRNA modifications and characterize these enzymes regarding their binding sites and functional partners are the next important steps toward a better understanding of the biological role of mRNA modifications in gene expression control of trypanosomatids.

## Conclusions

Our study sheds light on aspects of the functional relevance of chemical modifications in the transcriptome of *Leishmania mexicana*, demonstrating that the deletion of genes encoding the TRMT61A and NSUN2 orthologs disrupts normal cell differentiation and alters key biological pathways at the transcriptomic level. The distinct effects associated with each methyltransferase ortholog suggest that the m1A and m5C modifications may influence gene regulation via specific molecular mechanisms. Nevertheless, the convergent regulation of a subset of common target genes suggests potential crosstalk or functional convergence within a shared regulatory pathway, acting in a coordinated fashion to modulate gene expression. These findings advance our understanding of RNA-based regulatory networks in trypanosomatids, particularly regarding post-transcriptional gene expression in these protozoan pathogens.

## Supplementary Information

Additional file 1: Table S1. Sequences of the oligonucleotides used in the amplifications. Homologous sequence in capital letters and primer binding site in lower case letters. The lower case initial part of the sequence indicates the T7 RNA Pol promoter region, the target gene in capital letters and the lowercase final part indicates the Cas9 binding region.

Additional file 2: Fig. S1. Growth curves of promastigote stage of TRMT61A and NSUN2 knockout cell lines. Triplicate curves with cell density values plotted from daily counts, from moment 0 to 96 hours. Each cell line is represented with one respective color and shape, according to the graph legend.

Additional file 3: Table S2. Comparative analysis of gene expression between *Leishmania mexicana* strains T7/Cas9 vs WT.

Additional file 4: Fig S2. Principal component analysis (PCA) of gene expression profiles. (A) T7/Cas9 vs TRMT61A SKO, (B) T7/Cas9 vs NSUN2 2540 DKO, and (C) T7/Cas9 vs NSUN2 2170 DKO.

Additional file 5: Table S3. Complete list of the differentially expressed genes in the cell line containing a single knockout of *TRMT61A *gene.

Additional file 6: Fig S3. Comparative analysis of the RNA-seq reads coverage at the TRMT61A and NSUN2 *loci* in T7/Cas9 and mutant lines.

Additional file 7: Table S4. Complete list of the differentially expressed genes in the cell line containing a double knockout of *NSUN2 2540 *gene.

Additional file 8: Table S5. Complete list of the differentially expressed genes in the cell line containing a double knockout of *NSUN2 2170 *gene.

Additional file 9: Table S6. List of the RNA motifs identified with MEME suite tool.

## Data Availability

Sequence data that support the findings of this study have been deposited in the NCBI SRA with the primary accession code PRJNA1062791.

## Abbreviations

TRMT6: TRNA methyltransferase 6 non-catalytic subunit
TRMT61A: TRNA methyltransferase 61A
NSUN2: NOP2/Sun RNA methyltransferase 2
NAT10: N-acetyltransferase 10
SKO: Single knockout
DKO: Double knockout
BSD: Blasticidin
PAC: Puromycin
FTO: Fat mass and obesity-associated protein
RBM: RNA-binding motif protein
tRNA: Transfer RNA
mRNA: Messenger RNA
T7/Cas9: T7 RNA polymerase/CRISPR-Cas system
ALKBH: Alkb homolog
YTHDF: YTH domain-containing family
TET: Ten-eleven translocation
ALYREF: Aly/REF export factor
YBX: Box binding protein box
PABP: Poly (A) binding protein
HNRNPs: Heterogeneous nuclear ribonucleoproteins
IGF2BP2: Insulin-like growth factor 2 mRNA-binding protein 2
METTL3: Methyltransferase-like 3
DNMT2: DNA methyltransferase 2
CLIP-seq: Crosslinking and immunoprecipitation followed by sequencing
