# Supplementary figures and images for: Impact on the Leishmania mexicana transcriptome due to knockout of genes encoding orthologs of methyltransferases involved in m1A and m5C mRNA modifications
Source: Parasit Vectors. 2025 Jul 31;18:315. doi: 10.1186/s13071-025-06969-8 (PMC12315365; doi:10.1186/s13071-025-06969-8)

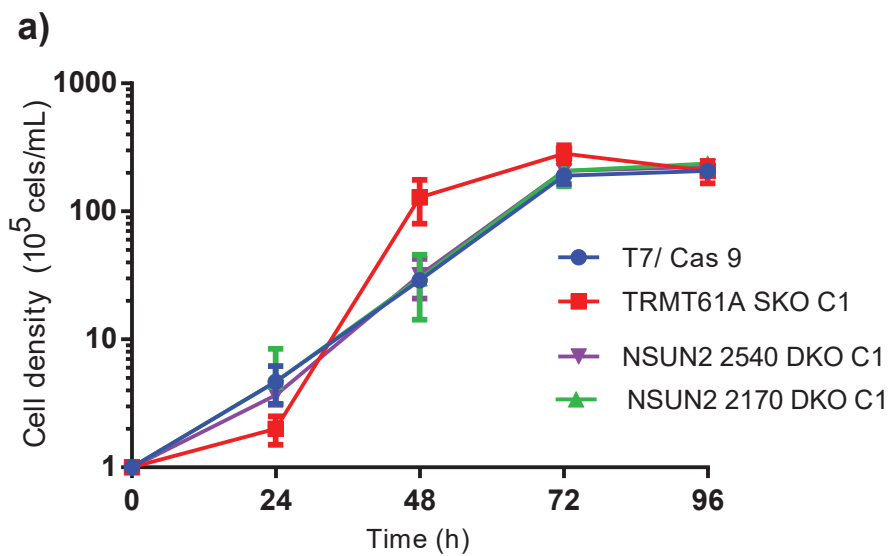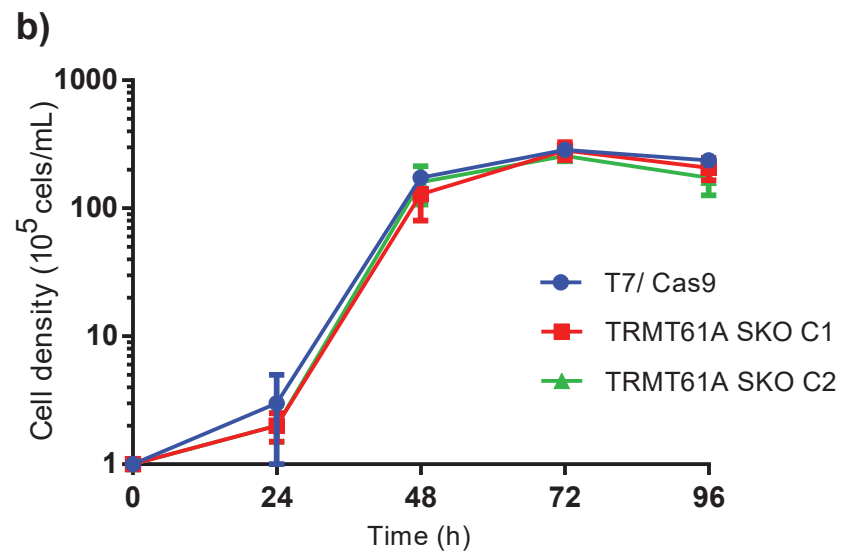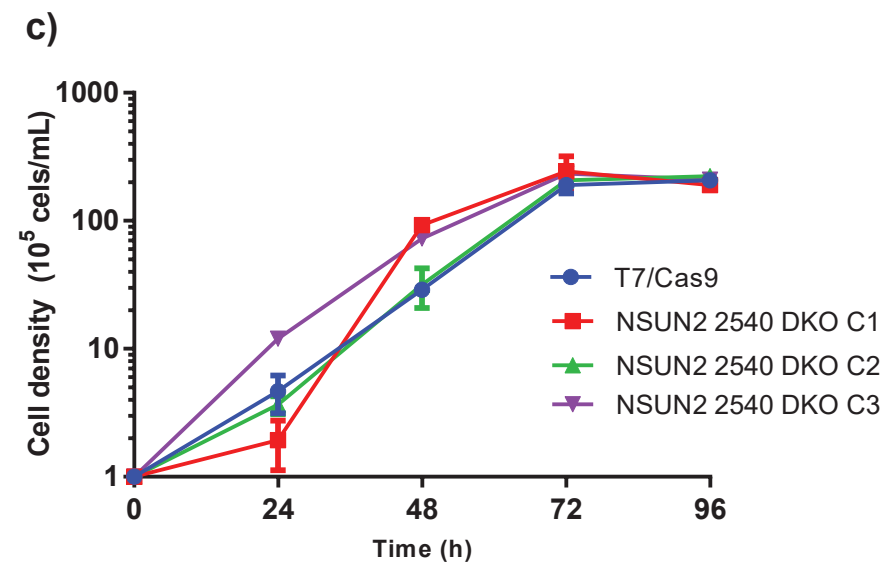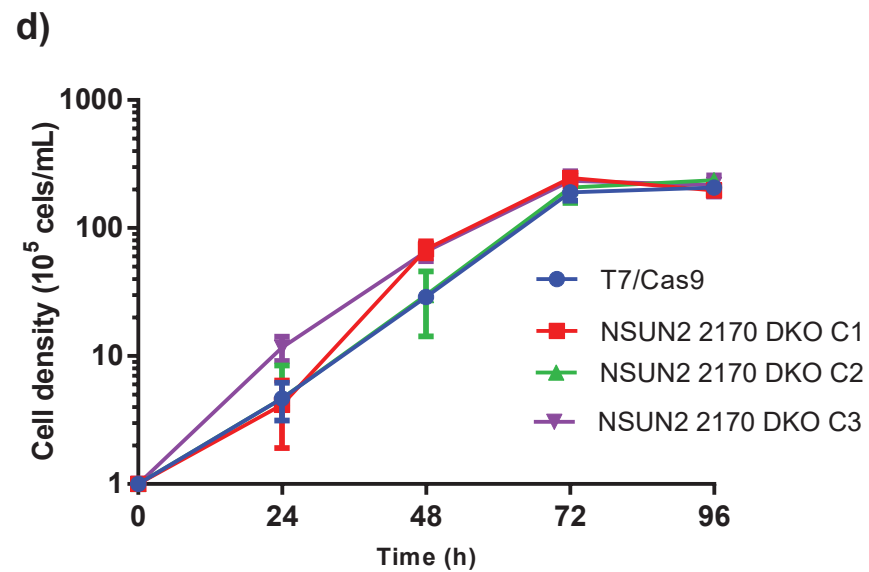

Supplement: Supplementary file 2 — Additional file 2: Fig. S1. Growth curves of promastigote stage of TRMT61A and NSUN2 knockout cell lines. Triplicate curves with cell density values plotted from daily counts, from moment 0 to 96 hours. Each cell line is represented with one respective color and shape, according to the graph legend. [file 13071_2025_6969_MOESM2_ESM.pdf]

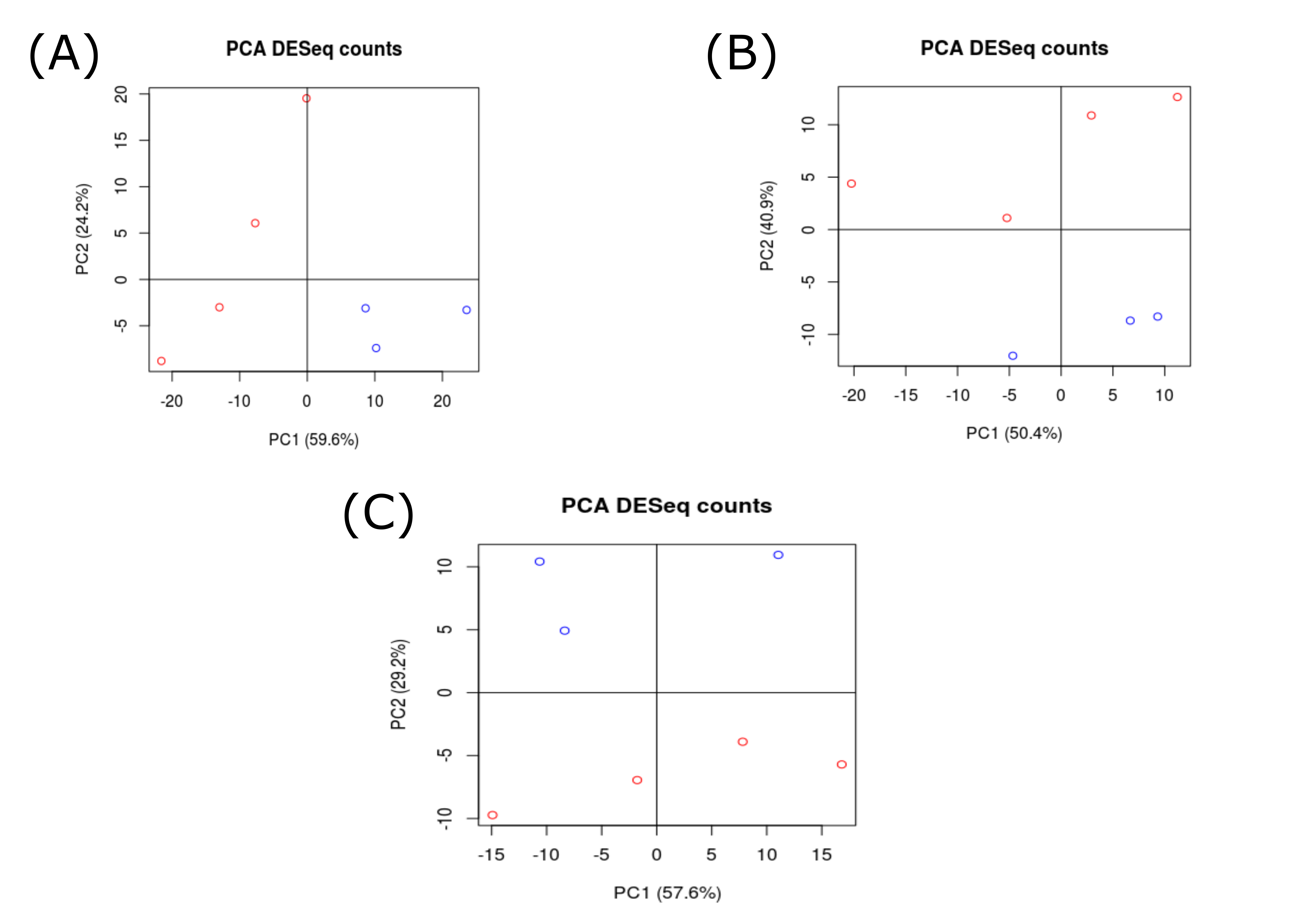

Supplement: Supplementary file 4 — Additional file 4: Fig S2. Principal component analysis (PCA) of gene expression profiles. (A) T7/Cas9 vs TRMT61A SKO, (B) T7/Cas9 vs NSUN2 2540 DKO, and (C) T7/Cas9 vs NSUN2 2170 DKO. [file 13071_2025_6969_MOESM4_ESM.tif]

Mean TPM by Sample and Gene with Standard Error

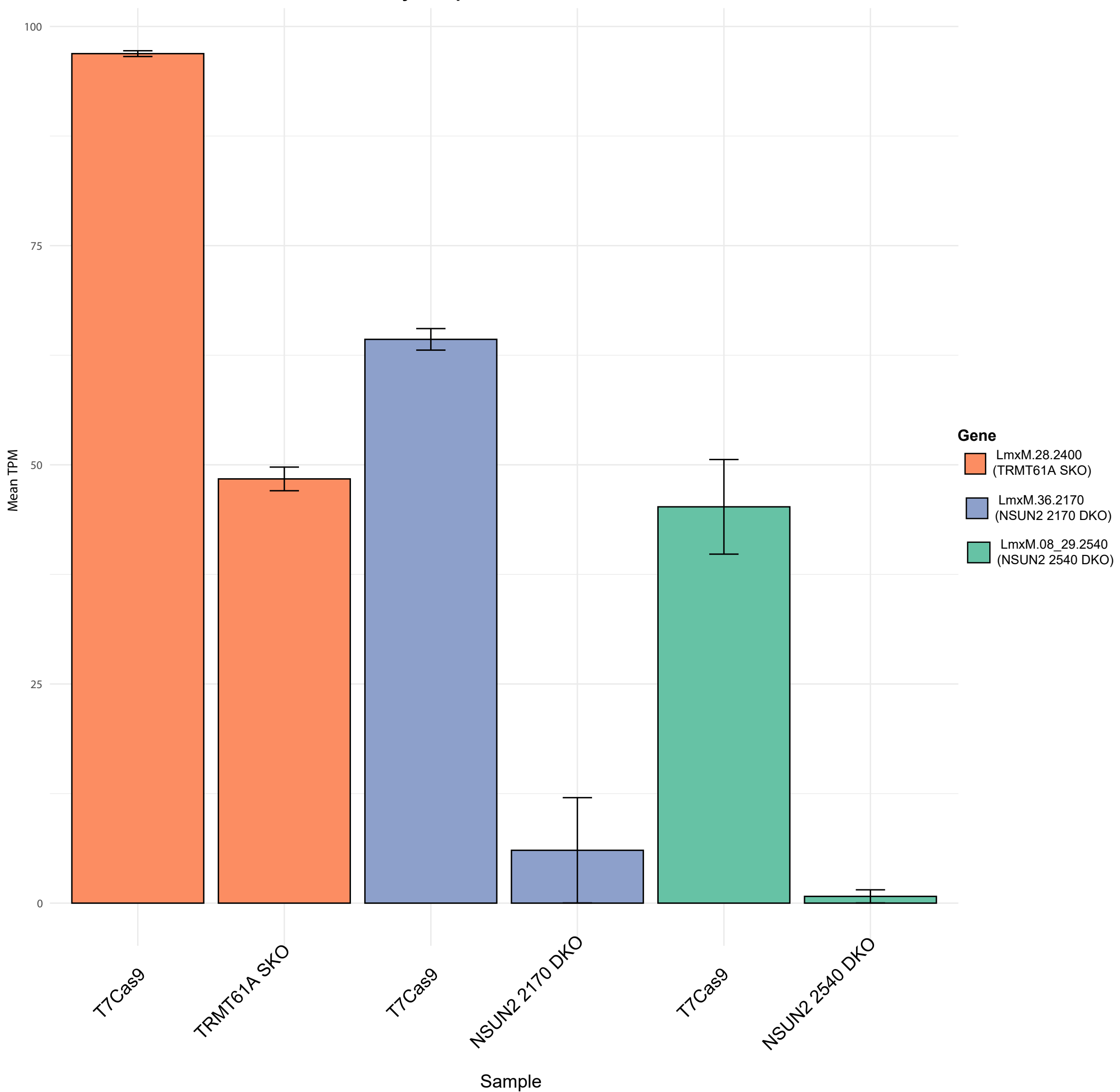

Supplement: Supplementary file 6 — Additional file 6: Fig S3. Comparative analysis of the RNA-seq reads coverage at the TRMT61A and NSUN2 loci in T7/Cas9 and mutant lines. [file 13071_2025_6969_MOESM6_ESM.pdf]
